# EZH2 upregulation by ERα induces proliferation and migration of papillary thyroid carcinoma

**DOI:** 10.1186/s12885-019-6306-9

**Published:** 2019-11-12

**Authors:** Liqiong Xue, Hongzhu Yan, Ying Chen, Qifa Zhang, Xin Xie, Xiaoying Ding, Xiaojing Wang, Zhongqing Qian, Feng Xiao, Zhiyi Song, Yijie Wu, Yongde Peng, Huanbai Xu

**Affiliations:** 1 Department of Endocrinology and Metabolism, Shanghai General Hospital, School of Medicine, Shanghai Jiao Tong University, Shanghai, China; 2 Department of Oncology, Shanghai East Hospital, Tongji University School of Medicine, Shanghai, China; 3 Department of Pathology, Seventh People’s Hospital of Shanghai University of Traditional Chinese Medicine, Shanghai, China; 4 Department of Urology, Department of Endocrinology and Metabolism, Shanghai Traditional Chinese Medicine-Integrated hospital, Shanghai university of Traditional Chinese Medicine, Shanghai, China; 5 Anhui Clinical and Preclinical Key Laboratory of Respiratory Disease, Bengbu Medical College, Bengbu, China

**Keywords:** Enhancer of zeste homolog 2, Estrogen receptor alpha, Proliferation, Migration, Papillary thyroid carcinoma

## Abstract

**Background:**

The incidence of papillary thyroid carcinoma (PTC) has been increasing worldwide in recent years. Therefore, novel potential therapeutic targets for PTC are urgently needed. Enhancer of zeste homolog 2 (EZH2), a methyltransferase belonging to PRC2, plays important roles in epigenetic silencing and cell cycle regulation. EZH2 overexpression has been found in several malignant tumor tissues, while its expression and function in PTC are largely unknown.

**Methods:**

Sixty-five cases of PTC tissue confirmed by pathology and 30 cases of normal thyroid tissue adjacent to PTC tissue were collected from patients undergoing surgical treatment, between February 2003 and February 2006. We investigated the clinic pathologic significance of EZH2 expression using Realtime-PCR and IHC in 65 human PTC tissues and 30 normal thyroid tissue samples. The EZH2 expression in human PTC cell lines (K1 and W3) and the normal thyroid follicular epithelial cell line Nthy-ori 3–1 was analyzed by Western blotting and Realtime PCR. The expressions of ERα and ERβ in cell lines were analyzed by Realtime PCR.The tumor cell biological behavior was evaluated by CCK8 assay, colony formation assay, transwell migration assay and xenograft tumors model.

**Results:**

Higher rate of EZH2 expression was found in PTC tissues than in normal thyroid tissues, EZH2 expression is associated with lymph node metastasis and recurrent. Inhibition of EZH2 in PTC cell lines downregulates cellular proliferation and migration. PTC is a disease with high incidence of female and E2-ERα upregulates EZH2 expression.

**Conclusions:**

These results suggest a potential role of EZH2 for the PTC growth and metastasis. As a novel therapy, a pharmacological therapy targeting EZH2 has full potential in treatment of PTC.

## Background

Papillary thyroid carcinoma (PTC) accounts for 70 to 80% of all thyroid cancers and is the most common type of thyroid cancer [1]. In recent years, with the rapid growth of the incidence of PTC, the associated diagnosis and treatment has brought great economic and psychological burden globally [2, 3]. The molecular mechanisms of PTC are terribly complicated, involving gene mutations and abnormal amplification, epigenetic modifications, abnormal protein ubiquitination and signaling crosstalk, to name only a few. Therefore, it is of great clinical significance to find more potential molecules in the treatment strategy of PTC.

Polycomb group (PcG) protein plays key roles in regulating cell proliferation and differentiation. As a member of the PcG family and the core catalytic component of the polycomb repressive complex 2 (PRC2), Enhancer of zeste homolog 2 (EZH2) acts by catalyzing trimethylation on histone 3 lysine 27 (H3K27me3) which results the silencing of its target genes [4]. More and more evidences show that EZH2 is involved in diverse fundamental cell processes, including cell proliferation and differentiation, cell cycle regulation and fate decision, tumorigenesis, cancer stem cell maintenance, and drug resistance [5–9]. Overexpression of EZH2 is positively correlated with tissue pathological grade and stage, metastasis, and poor survival in many types of solid tumors, including lung cancer, breast cancer, gastric cancer, prostate cancer, and melanoma [10–12]. Trimethylation of H3K27 is a crucial epigenetic label, and increased global levels of H3K27me3 are suggested to associate with poor prognosis [13]. EZH2 plays carcinogenic function through both PRC2-dependent and PRC2-independent activities [14, 15]. But EZH2 expression and function in carcinogenesis and tumor progression of PTC has not yet been clarified.

For this, we examined EZH2 expression level in clinical PTC tissue and found that it is higher in tumor tissues. Furthermore, we found that higher expression of EZH2 was significantly related to the aggressiveness and poor prognosis of PTC. Overexpression of EZH2 in PTC cell lines upregulates cellular proliferation and migration, which is regulated by E2-ERα signaling pathway. Our study suggests potential roles of EZH2 in the growth and metastasis of human PTC.

## Methods

### Human tissue samples

Sixty-five cases of PTC tissue confirmed by pathology and 30 cases of normal thyroid tissue adjacent to PTC tissue were collected from patients undergoing surgical treatment Shanghai General Hospital and Shanghai Seventh People’s Hospital, between February 2003 and February 2006. All participants did not receive any preoperative treatment. All the tissues were dissected, then immediately frozen in liquid nitrogen, and stored at − 80 degree °C for future treatment. The histological sections of samples were reviewed by two pathologiststogether to verify the diagnosis. The patients were divided to two group according to the EZH2 expression, high group means the EZH2 expression is higher than the median, and low group means the EZH2 expression is equal to or lower than median. This study was approved by the Medical Ethics Committee of Shanghai General Hospital and all the research works were carried out in accordance with the Helsinki declaration. All participants signed written informed consent before participating in this study.

### Cells, cell culture

Human PTC cell line K1 was purchased from the American Type Culture Collection(ATCC Catalogue No.92030501), and PTC cell line W3 was a kind gift from Dr. Robert Gagel (MD Anderson Cancer Center, University of Texas). Human thyroid cell line (human thyroid follicular epithelial) Nthy-ori 3–1 was purchased from the European Collection of Animal Cell Cultures (ECACC Catalogue No. 90011609). No further authentications were performed by the authors, except for the exclusion of mycoplasma infection. K1 cells, W3 cells and Nthyori 3–1 cells were cultured in DMEM-Ham’s F12-MCDB 105 (2:1:1) (Invitrogen), DMEM, and RPMI-1640 (Invitrogen) medium respectively, all supplemented with 10% fetal bovine serum (FBS) (Gibco), 100 μg/mL streptomycin, and 100 U/mL penicillin.

### RNA extraction and quantitative real time PCR (qPCR)

Total RNA was isolated from  tissue samples and cells using the TRIzol (Invitrogen), and reverse transcribed. Followed by qPCR with Power SYBR Green PCR Master Mix (Eppendorf), each gene relative expression levels were calculated and normalized to β-actin as an endogenous control using the 2-△△CT method. All reactions were performed in triplicates.

### Immunohistochemistry

Tissue specimens were fixed in 10% neutralized formalin and embedded in paraffin blocks. Sections were subjected to routine deparaffinization and rehydration. Antigen retrieval was performed by microwavingin 0.01 mol/L citrate buffer for 10 min. After inhibition of endogenous peroxidase activity for 20 min with methanol containing 3% hydrogen peroxide, sections were blocked with 2% BSA in PBS. After three PBS washes, the specimens were reacted overnight at 4 °C with EZH2 and ERα antibody (Abcam). The sections were then counterstained with hematoxylin and mounted. IHC staining was independently examined by two clinical pathologists who were unaware of the patient outcome. Interpretation and evaluation of IHC results was as described previously [16].

### Western blotting

The total proteins were separated by standard SDS-PAGE. Equal amounts of protein were transferred to a polyvinylidene difluoride membrane (Millipore), immunoblotted with first antibodies against ERα (Abcam) or ERβ (Abcam), and visualized with horseradish peroxidase–conjugated secondary antibodies. The GAPDH antibody was purchased from Sigma-Aldrich, and antibodies against p38 PK kinase, phospho (p)-p38 MAPK, ERK1/2 andphospho-ERK1/2were acquired from Cell Signaling technology.

### Cell proliferation assays

Cell proliferation was measured with Cell Counting Kit-8 (CCK-8) (Dojindo Laboratories). Cells were seeded in triplicate in 96-well plates at a density of 5000 cells/well. Each condition was repeated three times. All the cells were harvested at the designated times after treatment.

### Cell migration assay

Cells were suspended in serum-free medium at 5 × 10^5^ cells/mL and seeded into the upper chambers of Transwell chamber (Millipore). RPMI-1640 medium containing 10% FBS was added to the lower chamber. Cells were allowed to migrate for 12 h at 37 °C. The nonmigrating cells were gently removed from the upper surface of the membrane. The fixed and stained migrated cells, adherent to the lower surface of the membrane were photographed using an inverted light microscope and counted manually using 5 randomly selected areas. Each experiment was repeated three times.

### Animals and tumor model

Female nude mice (6–8 weeks) were purchased from Shanghai Laboratory Animal Center at the Chinese Academy of Sciences. Mice were housed in a specific pathogen-free facility at the Shanghai Jiao Tong University School of Medicine. All animal procedures were approved by the Animal Welfare & Ethics Committee of Shanghai Jiao Tong University School of Medicine. Five animals were used in each group. Mice were injected subcutaneously into the left flanks with 6 × 10^6^ K1 or W3 control or EZH2 knockdown cells suspended in PBS. Tumor volumes were estimated using the formula (length × width^2^)/2. Tumor were measured every 4 days.

### Statistical analysis

SPSS 19.0 software was used for statistical analysis. Relationship between staining intensity and Clinicopathology was assessed using χ^2^-test and two-sided Fisher’ exact test. All the data are expressed as means s.e.m. for at least three separate experiments, using an independent *t* test to perform comparisons of two independent groups.

## Results

### EZH2 is upregulated in clinical PTC tissue and cell lines

**Fig. 1:**
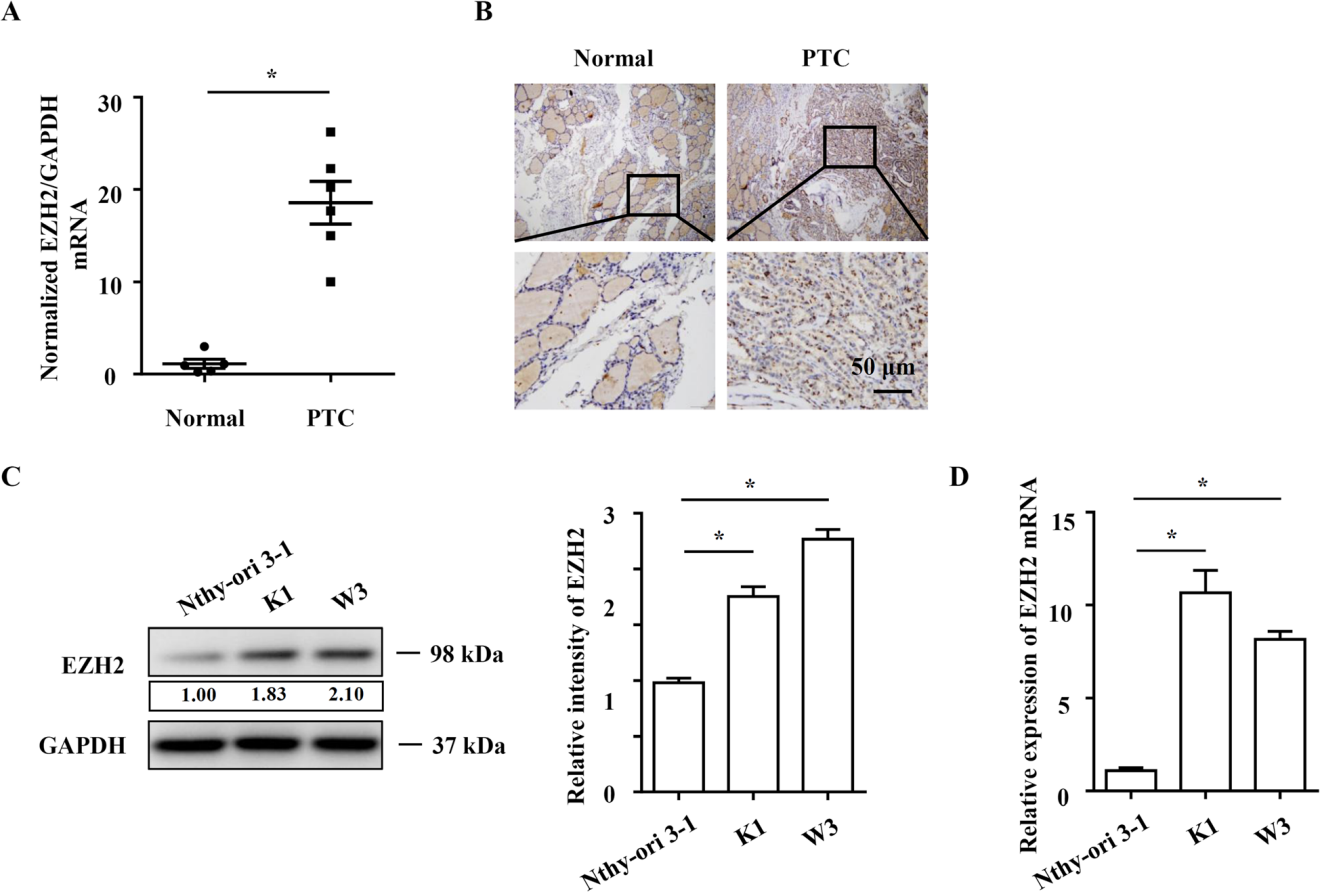
EZH2 is upregulated in clinical PTC tissues and cell lines. EZH2 expression was examined by Realtime-PCR (**a**) and IHC (**b**) in 65 PTC cancer samples and 30 normal thyroid tissues adjacent to cancer. Original magnification: × 100 (upper) and × 400 (lower). EZH2 protein and mRNA expression levels in two human PTC cell lines (K1 and W3) and the normal thyroid follicular epithelial cell line (Nthy-ori 3–1) were analyzed by Western blotting (**c**) and Realtime PCR (**d**). Data represent the mean ± S.E.M. *P < 0.05

To explore the EZH2 function in human PTC progression, we tried to study the association between its expression and clinicopathological features of PTC. We examined EZH2 expression in PTC tissue using Immunohistochemistry (IHC) staining and Real-time PCR. Expression of EZH2 is associated with lymph node metastasis (*p* = 0.0073) and recurrent (*p* = 0.0302) (Table 1). As shown in Fig. 1a, EZH2 mRNA level was significantly higher in PTC tissue than in paired normal thyroid tissue. The IHC staining result showed that EZH2 protein was expressed in 58% (38/65) of PTC tissues and in 10% (3/30) of paired normal thyroid tissues (Table 1, Fig. 1b). Meanwhile, EZH2 mRNA and protein expression levels were higher in human PTC cell line K1 and W3 than in normal thyroid follicular epithelial cell line Nthy-ori 3–1 (Fig. 1c and d). The results support EZH2’s potential role in the development and progress of PTC.

**Table 1:** Correlation of EZH2 expression with clinicopathologic feature in PTC

Clinicopathologic parameters	Case no.	EZH2 expression		*P*-value
		Low	High	
Total cases	65	27	38	
Age				0.4033
≤ 45	40	15	25	
> 45	25	12	13	
Gender				0.3751
Male	7	4	3	
Female	58	23	35	
Tumor size				0.8904
≤ 1 cm	18	10	8	
> 1 cm	47	27	20	
Extrathryoid extension				0.1899
No	59	23	36	
Yes	6	4	2	
Lymph node metastasis				0.0073
No	37	23	14	
Yes	28	8	20	
Recurrent				0.0302
No	59	27	32	
Yes	6	0	6

### EZH2 downregulation limits PTC cell proliferation and migration

**Fig. 2:**
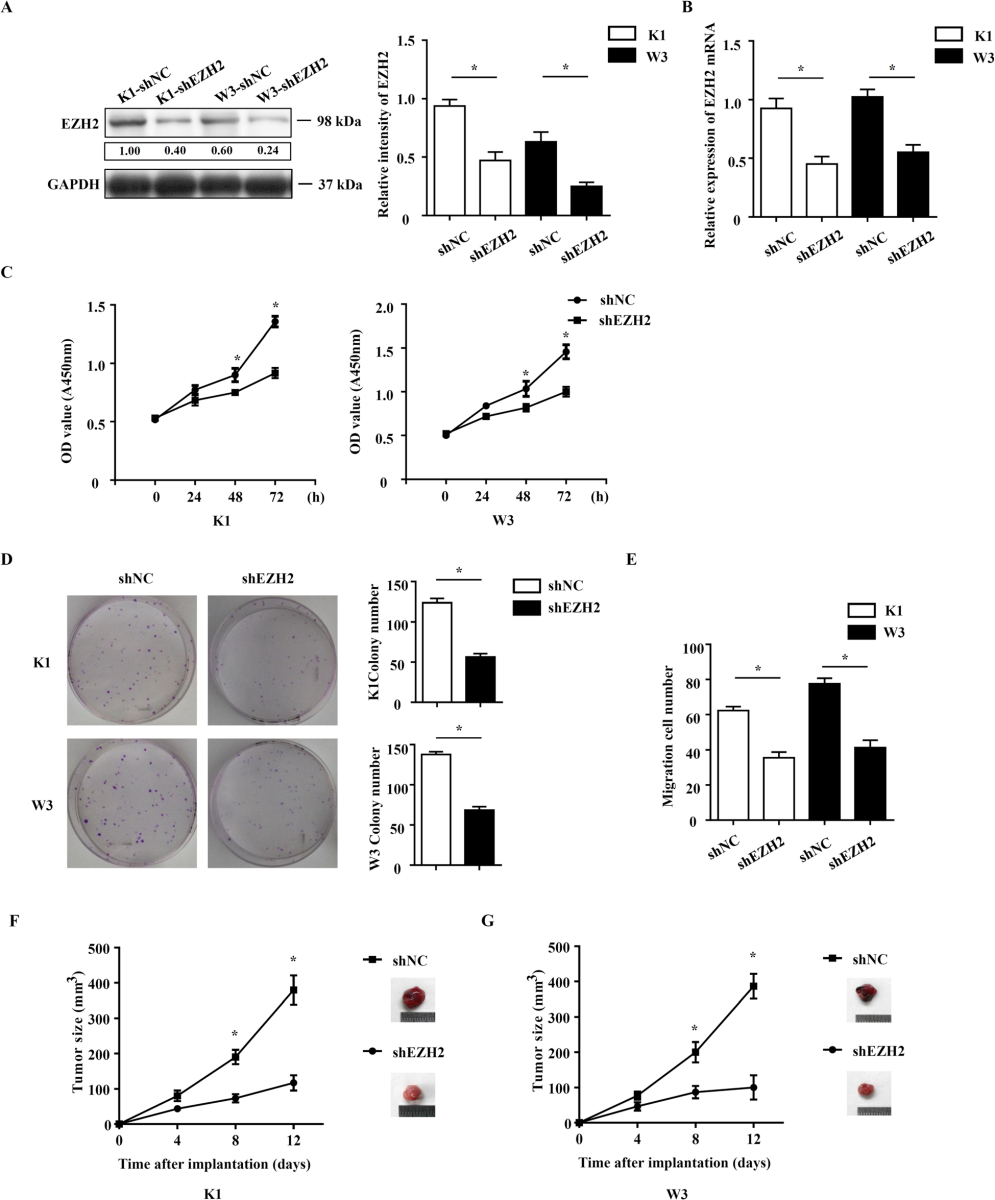
EZH2 downregulation limits PTC cell proliferation and migration. **a** and **b** Western blot analysis and Realtime PCR of EZH2 expression in K1 and W3 cell infected with EZH2 lentivirus (indicated as shEZH2) and control lentivirus (indicated as shNC). **c** CCK8 assay. **d** Colony formation assays. **e** Transwell migration assay. **f** and **g** Xenograft model in nude mice. Tumor were measured every four days. Data represent the mean ± S.E.M. *P < 0.05

To characterize the effect of EZH2 on cell proliferation, and migration, which are required for tumorigenesis and metastasis, we knocked down EZH2 in K1 and W3 cells using a short hairpin RNA (shEZH2), with scrambled shRNA as control (shNC). Western blotting and Real time PCR were used to confirm Knockdown efficiency (Fig. 2a and b). The CCK-8 assay showed that EZH2 knockdown reduced the viability of K1 and W3 cell compared with controls (Fig. 2c). EZH2 knockdown also decreased colony formation in K1 and W3 cells (Fig. 2d). Subsequently, we used Transwell assay to test whether EZH2 regulates tumor cell migration. These data showed that EZH2 knockdown strongly inhibited PTC migration capacities (Fig. 2 E). Next, we examined the effect of EZH2 on PTC by using xenograft tumors model with control or EZH2 knockdown cell lines. The result showed that when EZH2 expression was inhibited, tumors grew significantly slower that the control group (Fig. 2f and g). Collectively, our data indicated that EZH2 knockdown suppresses proliferation of PTC cells.

### Estrogen upregulated EZH2 to promote the PTC cell proliferation and migration

**Fig. 3:**
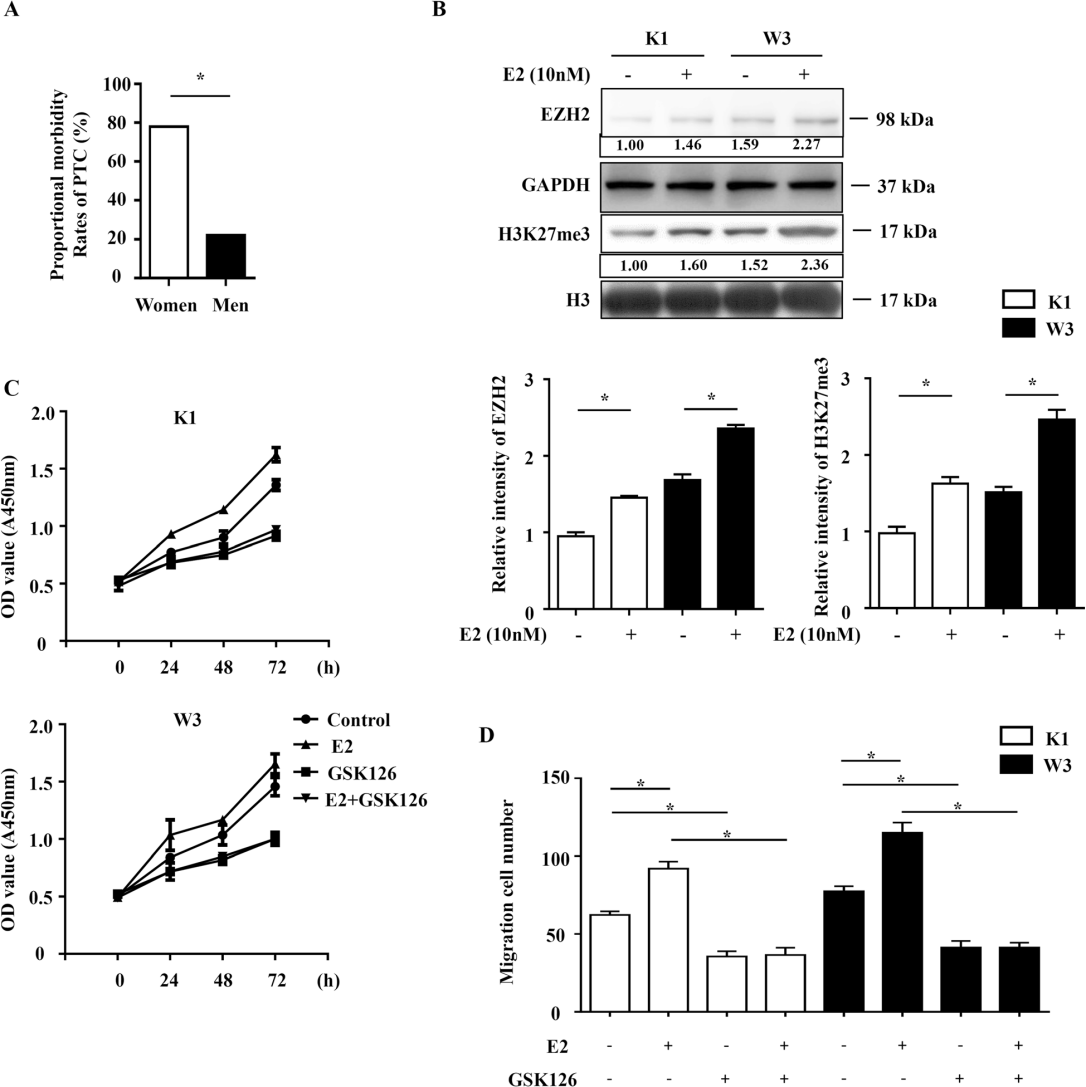
Estrogen upregulated EZH2 to promote the PTC cell proliferation and migration.**a** The proportional morbidity rates of PTC in female and male patients with PTC. **b** K1 and W3 cell lines were treated with 10 nM E2 for 24 h, and then were analyzed by western blotting. **c** and **d** K1 and W3 cell lines were treated with 10 nM E2 and 2 μM GSK126, and then were analyzed by CCK8, Colony formation assays and transwell migration assay. Data represent the mean ± S.E.M. *P < 0.05

Previous study and our result showed that the incidence of PTC in females was higher than that in males (Fig. 3a), suggesting that an estrogen-related signaling pathway might have important roles in PTC development. Treatment with E2 led to a significantly upregulation of EZH2. Interestingly, the level of H3K27me3 was also increased after E2 treatment (Fig. 3b), which due to the increased level of EZH2 in PTC cell. E2 treatment increased proliferation and migration in both K1 and W3 cells. Besides, the specific EZH2 inhibitor GSK126 can reversed the increase of proliferation and migration mediated by E2 (Fig. 3c and d), indicating that EZH2 was the target of E2. These data suggest that E2 upregulated EZH2 expression to promote the PTC development.

### ERα contributes to the increase of EZH2 in PTC cells

**FIG. 4:**
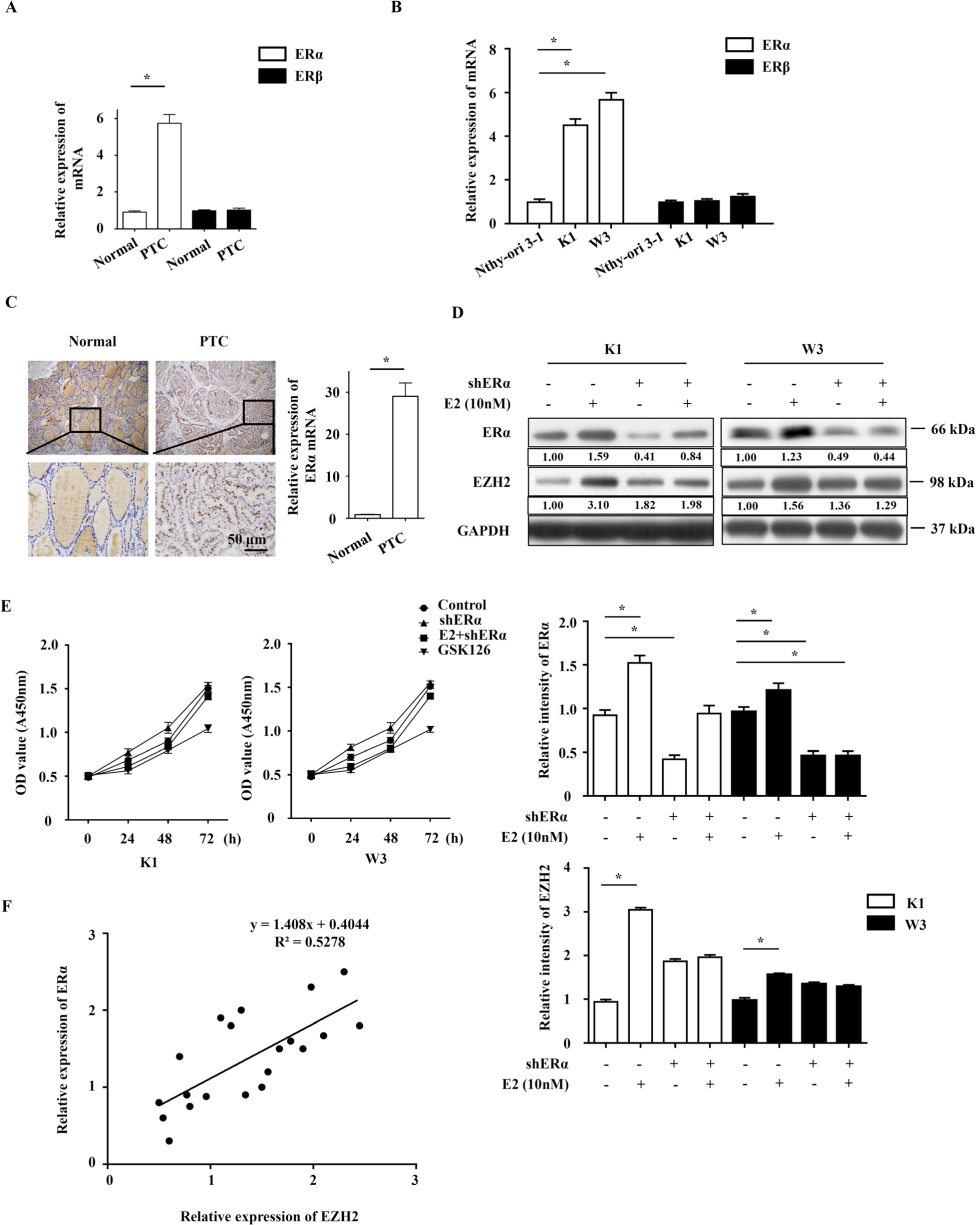
ERα contributes to the increase of EZH2 in PTC cells. **a** ERα and ERβ expression levels were examined by Realtime-PCR in PTC and paired normal thyroid tissues. **b** ERα and ERβ expression in K1, W3 and Nthy-ori 3–1 cells were analyzed by Realtime PCR. **c** ERα expression was examined by IHC. Original magnification: × 100 (upper) and × 400 (lower). **d** Western blot analysis of EZH2 expression in K1 and W3 cells infected with shERα in the presence or absence of 10 nM E2. **e** K1 and W3 cell lines infected with shERα in the presence or absence of 10 nM E2 and 2 μM GSK126, and then were analyzed by CCK8. **f** Relationship of relative EZH2 and ERα mRNA expression. Data represent the mean ± S.E.M. *P < 0.05

Two structurally related receptors ERα and ERβ were reported to bind E2 as ligand. However, we found that expression of ERα, but not ERβ, was up-regulated in the PTC patient tissues and cells lines, indicating the potential critical role of ERα in PTC development (Fig. 4a and b). We also detected the ERα expression in PTC by IHC, establishing that ERα was up-regulated in PTC samples (Fig. 4c). When ERα was knockdown, E2 couldn’t upregulate the expression of EZH2 anymore (Fig. 4d). Compared with GSK126 treatment, ERα knockdown plus E2 treatment had less effect on cell proliferation, which may due to the EZH2 expression levels (Fig. 4e). Furthermore, we found that expression of EZH2 in human PTC samples was positively related to that of ERα with an efficiency of R^2^ = 0.5278 (Fig. 4f). Collectively, these data suggest that E2 upregulates the expression of EZH2 through ERα in the PTC cells.

## Discussion

In this study, EZH2 was found highly expressing in PTC tissues and cell lines, suggesting that its expression may contribute to the development and progression of PTC as an oncogene. These results are consistent with previous data demonstrating of EZH2 overexpression in other types of cancer such as breast, prostate, and pancreatic cancers [17]. By Fisher’ exact test analysis, our study indicates EZH2 expression is highly correlated with PTC lymph node metastasis and recurrent, suggesting that EZH2 might contribute to PTC development and progression.

Our investigation identified EZH2 as a positive regulator of PTC progression. Taking advantage of knockdown assay, our data support that EZH2 plays a critical role in PTC growth, as well as highly metastatic. Similar observation have been reported in tumor samples, such as lung cancer, pancreatic cancer, prostate cancer, and breast cancer. Unfortunately, there is no significant difference of mortality in EZH2-high and EZH2-low patient groups. It is likely due to the fact that the malignancy of PTC is low and the time of follow-up should be prolonged. Women are affected more frequently than men particularly during fertile period of women compared with men of the same age [18]. Estrogens are involved in the growth and differentiation of the normal mammary gland [19]. It has been found that estrogen can increase the growth, progression and metastasis of PTC [20, 21], and it is no wonder that estrogen exerts a more important role in the pathogenesis of PTC in young women (under 25 years of age) than in women 30 years and older [22]. Treatment with E2 could upregulate the levels of EZH2 in PTC cell line as well as its methyltransferase activity significantly. Besides, the specific EZH2 inhibitor GSK126 can reverse the increase of proliferation and migration mediated by E2, indicating that EZH2 was the target of E2.

Estrogen-mediated changes at the cellular level are mostly mediated via its receptors, ERα and ERβ. Our study found that E2 interacted with ERα upregulated EZH2. ERα, encoded by the gene *ESR1* in human, is a nuclear receptor that has a key role in cell proliferation ad differentiation. ERα was overexpressed in the PTC patients and positively correlated with the levels of EZH2 expression. Previous study has showed that ERα could directly regulate EZH2 expression when recruited to its promoter [23]. However how EZH2 regulation by ERα is still unknown. Also, ERα can be targeted with specific inhibitors or tamoxifen. Actually, our previous study has demonstrated that miR-219–5p dramatically inhibited PTC cell growth and migration by targeting ERα [24]. As EZH2 is regulated by ERα, EZH2 could be another potential target for PTC therapy. Exactly, EZH2 is the first PcG gene verified to be regulated by miRNA. miR-26 and miR-101 reduce cellular EZH2 level through targeting the 3′ untranslated region of EZH2 messenger RNA [25, 26]. Besides, a subset of miRNA in tumor tissue, including miR-181a, miR-181b, miR-200b, miR-200c, and miR-203, are transcriptionally silenced by PRC2 [27]. The relationship between EZH2 and miR-219-5p is worthy to further explore in the future.

## Conclusion

In summary, the current study demonstrated that EZH2 was overexpressed in PTC tissue and EZH2 downregulated PTC cells proliferation and migration, which was partly mediated by E2-ERα signal pathway. Furthermore, we found that higher expression of EZH2 was linked to lymph node metastasis and recurrent. Thus, our results indicated that EZH2 is critical for the progression of PTC, epigenetic therapy pharmacologically targeting EZH2 through specific inhibitors may constitute a new therapeutic method for PTC.

## Data Availability

The datasets used and/or analyzed during the current study are available from the corresponding author on reasonable request.
